# HLA-G Polymorphisms Associated with HIV Infection and Preeclampsia in South Africans of African Ancestry

**DOI:** 10.1155/2020/1697657

**Published:** 2020-06-10

**Authors:** Wendy N. Phoswa, Veron Ramsuran, Thajasvarie Naicker, Ravesh Singh, Jagidesa Moodley

**Affiliations:** ^1^Discipline of Obstetrics and Gynecology, Nelson R. Mandela School of Clinical Medicine, University of KwaZulu-Natal, Durban, South Africa; ^2^KwaZulu-Natal Research Innovation and Sequencing Platform (KRISP), School of Laboratory Medicine and Medical Sciences, University of KwaZulu-Natal, Durban, South Africa; ^3^Centre for the AIDS Programme of Research in South Africa (CAPRISA), Durban, South Africa; ^4^Optics and Imaging Centre, University of KwaZulu-Natal, Durban, South Africa; ^5^Department of Microbiology, National Health Laboratory Services, KwaZulu-Natal Academic Complex, Inkosi Albert Luthuli Central Hospital, Durban, South Africa; ^6^Women's Health and HIV Research Group, University of KwaZulu-Natal, Durban, South Africa

## Abstract

**Objectives:**

HLA-G, part of the major histocompatibility complex (MHC), is associated with the risk of developing preeclampsia (PE). In this study, we determined the contribution of specific HLA-G polymorphisms on the risk of developing preeclampsia in HIV-infected and uninfected South Africans of African ancestry.

**Methods:**

One hundred and ninety-three women of African ancestry were enrolled (74 HIV-uninfected normotensive, 60 HIV-infected normotensive, 34 HIV-uninfected, and 25 HIV-infected preeclamptics). Sanger sequencing of the untranslated region was performed to genotype six SNPs, i.e., 14 bp Ins/Del of rs66554220, rs1710, rs1063320, rs1610696, rs9380142, and rs1707).

**Results:**

For rs66554220, we have the following results: (a) based on pregnancy type—the Ins/Ins and Del/Ins genotype frequency was higher in preeclampsia (PE) compared to normotensive pregnancies (Ins/Ins *vs*. Del/Ins, *P* = 0.02^∗^: OR (95%CI) = 13.44 (0.7222–249.9); Del/Del *vs*. Del/Ins, *P* = 0.03^∗^: OR (95%CI) = 2.95 (1.10–7.920)); (b) based on HIV status—the Ins/Ins showed both genotypic and allelic association with HIV infection. HIV-infected PE has higher Ins/Ins genotypic and allelic frequencies compared to HIV-uninfected PE (Ins/Ins *vs*. Del/Ins, *P* = 0.005^∗∗^: OR (95%CI) = 21.32 (1.71–4.17); Ins, *P* = 0.005^∗∗^; OR (95%IC) = 21.32 (1.71–4.17)). For rs1707, we have the following results: (a) based on pregnancy type—there were CT genotypic frequencies in PE, more especially LOPE compared to normotensive pregnancies (TT *vs*. CT, *P* = 0.0092^∗∗^: OR (95%CI) = 5.(1.39 − 25.64)), and no allelic association was noted; (b) based on HIV status—CT was higher in HIV-infected LOPE compared to uninfected LOPE (TT *vs*. TC, *P* = 0.0006^∗∗∗^: OR (95%CI) = 40.00 (2.89 − 555.1)). For rs1710 and rs1063320, no significant differences in the genotype and allele frequencies were noted based on pregnancy type and HIV status. For rs9380142, we have the following results: (a) based on pregnancy type—no significant differences were noted between normotensive compared to PE pregnancies; (b) based on HIV status—AA genotypes occurred more in the HIV-infected PE group (AA *vs*. GG, *P* = 0.02^∗^: OR (95%CI) = 13.97 (0.73 − 269.4)), while A allelic frequency occurred more in HIV-infected PE, especially LOPE compared to uninfected groups (A *vs*. G, *P* = 0.0003^∗∗∗^: OR (95%CI) = 10.72 (2.380 − 48.32); *P* = 0.02^∗^: OR (95%CI) = 9.00 (1.07 − 75.74)). For rs1610696, we have the following results: (a) based on pregnancy type—genotypic and allelic frequencies of CC were higher in PE compared to normotensive pregnancies (CC *vs*. GG, *P* = 0.0003^∗∗∗^: OR (95%CI) = 31.87 (1.861 − 545.9); C, *P* = 0.0001^∗∗∗^: OR (95%IC) = 21.91 (2.84 − 169.0)); (b) based on HIV status—GG frequencies were higher in the HIV-infected PE more especially LOPE groups (GG *vs*. GC, *P* = 0.02^∗^: OR (95%CI) = 16.87 (0.81 − 352.1); GG *vs*. CC, *P* = 0.0001^∗∗∗^: OR (95%CI) = 159.5 (13.10 − 1942)).

**Conclusion:**

Selected HLA-G 14 bp polymorphisms (Ins/Ins) and genotypic and allelic differences in rs9380142, rs1610696, and rs1707 are associated with the pathogenesis of preeclampsia in HIV-infected South African women of African ancestry. More genetic studies evaluating the association between preeclampsia and HIV infection are needed to improve diagnosis and antenatal care.

## 1. Introduction

Preeclampsia (PE) is a human-specific multisystemic disorder affecting 3–17% of pregnancies worldwide [1]. The diagnosis of PE is made clinically in the presence of new-onset hypertension (systolic and diastolic blood pressure of ≥140/90 mmHg) and proteinuria (≥300 mg in a 24-hour urine collection) after the 20th week of pregnancy [1, 2]. Preeclampsia may be classified by gestational age into early- (<33 weeks + 6 days; EOPE) or late- (>34 weeks + 0 days; LOPE) onset PE [2]. Preeclampsia is characterised by significant maternal, foetal, and neonatal morbidity and mortality worldwide but particularly in low- and middle-income countries [3].

Although the exact aetiology is unknown [4], it is thought to develop as a result of placental maladaptation due to impaired uterine spiral artery remodelling [5]. In normal placentation, the spiral arterioles are transformed into wide-bore channels that enable adequate blood supply to the developing foetus. In PE, the trophoblast invasion is deficient with a lack of physiological transformation of myometrial spiral arteries. Although the actual reason for the poor cytotrophoblast invasion remains unknown, both genetic and immune responses are thought to play a role [6].

There are conflicting data on the influence of HIV infection on the incidence of PE. Prevalence data on PE development in HIV-infected pregnancies are contradictory [4, 7]. Furthermore, it has been reported that HIV treatment regimen may predispose women to the development of PE [4]. Thus, the actual role of HIV infection in the pathophysiology of PE still needs to be established.

Notably, a balanced maternal immune response is needed for tolerance of the developing foetus to prevent spontaneous miscarriages. The human leukocyte antigen (HLA-G) plays a critical role in the maintenance of maternal immune response during pregnancy via the reduction of immune attacks raised against the semiallogeneic conceptus [8, 9]. The HLA-G antigen is primarily expressed by the extravillous trophoblast cells lining the placenta [10, 11] and is also involved in modulating immune responses in the context of vascular remodelling [12–14]. It also protects the developing foetus via the inhibition of cytotoxic CD8+ T cells as well as through natural killer (NK) cell activation [15, 16]. HLA-G also helps to prevent the proliferation of allospecific CD4+ T cells and regulate antigen-presenting cells (APCs) [17]. Moreover, it promotes differentiation of myeloid and T regulatory cells to enable maternal tolerance of the foetus during pregnancy [18].

Studies have shown that polymorphisms within the HLA-G region are associated with several disorders, including PE, recurrent spontaneous miscarriage, autoimmune diseases (lupus erythematosus, multiple sclerosis, and rheumatoid arthritis) [19], and pemphigus vulgaris [20]. An insertion of a 14-base pair (bp) sequence in the HLA-G 3′ UTR elicits a downregulation of HLA-G expression at the foetal-maternal interface with consequential decline in tolerance of the semiallogenic foetus. HLA-G 14 bp insertion/deletion polymorphism has also been shown to be associated with the risk of recurrent miscarriages [21]. The 14 bp insertion polymorphism (rs6655422) is also linked with susceptibility to PE development [3, 22, 23]. HLA-G∗01:01:03 and the HLA-G 01:05N null allele have been reported to play a role in PE development. Of note, studies have reported a significant increase of the 14 bp insertion polymorphism, HLA-G∗01:01:03, and HLA-G 01:05N null allele in PE in comparison to normal pregnancies [24–31]. Contradictory reports have also shown that a variation of SNPs within the HLA-G 3′ UTR, rs1710, rs1063320, rs1610696, rs9380142, and rs1707 may or may not be associated with PE development [18, 30, 32]. Moreover a 14 bp polymorphism and the C to G substitution in rs1063320 may influence the development of PE development in primiparas [30]. Also, a C/G polymorphism in rs9380142 has been associated with the stability of mRNA and may impact the expression of sHLA-G [32].

Alteration in the circulating levels of HLA-G has been shown in PE, suggesting its involvement in the development of this disorder [33–36]. Studies performed in India, Germany, Poland, and Iran have reported a strong association of the 14 bp insertion polymorphism (rs6655422) with PE development [30, 35]. However, there is a paucity of data on the 14 bp polymorphism in South Africans of African ancestry who develop PE. Hence, this study seeks to determine the association of HLA-G 14 bp polymorphism and genotypic and allelic frequencies of SNPs rs1710, rs1063320, rs1610696, rs9380142, and rs1707 with PE development. Despite immune maladaptation being implicated in the development of PE, the frequency of PE was also reported to be increased by African American ethnicity [37, 38] and immunosuppressive conditions, such as human immune deficiency virus (HIV) infection and acquired immunodeficiency syndrome (AIDS) [39].

South Africa has high rates of HIV infection (13.5% of total population) [40], and the KwaZulu-Natal province is the epicentre of HIV infection, with 40% HIV infection among pregnant women [7]. The current recommended treatment for HIV infection in pregnant and nonpregnant women is highly active antiretroviral therapy (HAART) [39]. The use of HAART in pregnancy is important for the prevention of mother-to-child transmission by several mechanisms, including lowering maternal antepartum viral load and preexposure and postexposure prophylaxis of the infant [41]. Nonetheless, the use of HAART during HIV infection has improved normal lifespan and turned this deadly disease into a chronic manageable condition [42]. However, some studies show that PE and foetal death have increased sharply in HIV-infected pregnant women receiving HAART [15, 43]. In this study, we investigated whether there was a genetic association of the six HLA-G gene polymorphisms with the risk of preeclampsia development in HIV-infected and uninfected South African women of African ancestry.

## 2. Materials and Methods

### 2.1. Study Population and Sample Collection

Institutional ethical and hospital regulatory permissions were obtained for the study (Biomedical Research Ethics Committee, University of KwaZulu-Natal, South Africa; BCA338/17). After written consent was obtained, preeclamptic (PE) and normotensive (N) HIV-infected and uninfected pregnant women were recruited at a public health care hospital in South Africa. Preeclampsia was defined as new-onset blood pressure of ≥140/90 mmHg taken on two occasions 4 hours apart and at least 1+ proteinuria measured by urinary dipstick. Normotensive pregnant participants were defined as those with a blood pressure of ≤120/80 mmHg and without evidence of proteinuria [44]. Early-onset preeclampsia (EOPE) was defined as new-onset blood pressure of ≥140/90 mmHg taken on two occasions 4 hours apart and at least 1+ proteinuria at gestation age of <33 weeks + 6 days, and late-onset preeclampsia (LOPE) was defined as new-onset blood pressure of ≥140/90 mmHg taken on two occasions 4 hours apart and at least 1+ proteinuria at gestation age of >34 weeks + 0 days [2]. The relevant data of all research participants were obtained from their maternity case records. HIV testing was done after counselling using a rapid point-of-care test kit initially, as is the standard of care in South Africa. To maintain ethnographic and anthropometric consistency, all patients recruited were of African ancestry and resident in the same geographical location. All participants were nonsmokers and nonconsumers of alcohol or recreational drugs, and all HIV-infected participants were on highly active antiretroviral therapy (HAART: tenofovir, emtricitabine, and efavirenz) as per South African national HIV guidelines at the time of the study [45]. Women with chronic medical conditions were excluded from the study.

### 2.2. Genomic DNA Extraction

Genomic DNA was extracted using the Thermo Fisher Scientific GeneJET Whole Blood Genomic DNA Purification Mini Kit (Thermo Fisher Scientific) from 500 *μ*l of whole blood. After extraction, the samples were stored at -20°C until genotyping analysis.

### 2.3. Amplification and Sequencing of HLA-G Gene

Polymerase chain reaction (PCR) was used to amplify the DNA sequences in a 20 *μ*l final reaction volume, using Phusion High-Fidelity DNA Polymerase (catalogue number: F5305). Final concentration of the forward and reverse primer was 10 pmol. The reactions were carried out in a SimpliAmp Thermal Cycler (Thermo Fisher Scientific). The following thermal cycler conditions were used: initial denaturation of 98°C for 30 seconds; followed by 35 cycles of 98°C for 30 seconds, 65°C for 30 seconds, and 72°C for 30 seconds; and a final extension of 72°C for 5 minutes. The PCR products (5 *μ*l) plus 1 *μ*l of loading dye were ran on gel.

After amplification using PCR, Sanger sequencing was performed as per the manufacturer's instructions (Thermo Fisher Scientific). The following primers were used: forward primer—5′-GTGATGGGCTGTTTAAAGTGTCAC-3′ (1.0 *μ*M) and reverse primer—5′-ATTGAAAGAGACCTGGAAGGAGGG-3′ (1.0 *μ*M). The obtained sequencing data were compared to the reference sequences (Hg37) with the aid of the Mutation Surveyor Software (SoftGenetics).

### 2.4. Statistical Analysis

The obtained genotypes were described using frequencies and percentages. The Hardy-Weinberg equilibrium (HWE) test was used to check for conformance to observed frequencies of the genotypes. The Chi-squared test or Fisher's exact test was used were suitable to compare data from the different subgroups. Odds ratios (OR) and 95% confidence interval (CI) were used to show the level of association for categorical data, and Wilcoxon's rank-sum tests were used for numeric data. Demographic data was analyzed using the GraphPad Prism 5 software (GraphPad Software, San Diego, CA, USA). A *P* value < 0.05 was considered statistically significant.

## 3. Results

### 3.1. Clinical Characteristics of Participants

Table 1 provides a summary of the clinical demographics of the study population. As expected, systolic and diastolic blood pressures (BP) differed between the normotensive and PE groups (*P* ≤ 0.0001). Similarly, gestational age was statistically different between the normotensive pregnant and PE groups (*P* < 0.001 each; two-sample Wilcoxon's rank-sum (Mann-Whitney's) test). There were no significant differences in maternal weight (*P* = 0.1316), maternal height (*P* = 0.6761), BMI (*P* = 0.0638), and maternal age (*P* = 0.9574) between normotensive versus EOPE versus LOPE groups.

### 3.2. Genetic Associations

The six HLA-G gene polymorphisms (14 bp (rs66554220), SNP 3022 (rs1707), SNP 3029 (rs1710), SNP 3161 (rs1063320), SNP 3206 (rs9380142), and SNP 3215 (rs1610696)) were tested for associations with HIV disease and PE using a cohort of South Africans of African ancestry. The cohort included HIV-infected and uninfected controls; in addition, there were normotensive and PE groups. Comparisons were performed *based on pregnancy type*, i.e., normotensive *vs*. preeclamptic groups, and *based on HIV status*, i.e., HIV-uninfected normotensive *vs*. HIV-infected normotensive, HIV-uninfected preeclamptic *vs*. HIV-infected preeclamptic, HIV-uninfected early-onset preeclamptic *vs*. HIV-infected preeclamptic, and HIV-uninfected late-onset preeclamptic *vs*. HIV-infected late-onset preeclamptic across all SNPs.

### 3.3. Genotypic and Allelic Associations of HLA-G 14 bp Ins/Del (rs66554220) with HIV and Preeclampsia

The genotypic frequencies across HIV-infected and uninfected individuals for HLA-G 14 bp Ins/Del showed no significant associations within the normotensive group (Table 2). However, comparing the genotypic frequencies of HIV-infected and uninfected donors from the PE group revealed a significant association (Del/Del *vs*. Ins/Ins, *P* = 0.004^∗∗^: OR (95%CI) = 25.13 (1.24–509.5); Ins/Ins *vs*. Del/Ins, *P* = 0.005^∗∗^: OR (95%CI) = 21.32 (1.71–4.17)) (Table 2). Furthermore, in the PE group, when comparing HIV status within EOPE, a significant difference in the genotypic frequencies of Del/Del was observed between HIV-uninfected EOPE and HIV-infected EOPE (Del/Del *vs*. Ins/Ins, *P* = 0.01^∗^: OR (95%CI) = 20.09 (0.93–433.1)) (Table 2). Individuals that were LOPE showed no significant differences. In the third group, normotensive *vs*. preeclamptic, Ins/Ins (9 HIV-uninfected normotensives and 0 HIV-uninfected PE) and Del/Ins (32 HIV-uninfected normotensives and 20 HIV-uninfected PE) were significant between the HIV-uninfected normotensives and HIV-uninfected PE (Ins/Ins *vs*. Del/Ins, *P* = 0.02^∗^: OR (95%CI) = 13.44 (0.7222–249.9); Del/Del *vs*. Del/Ins, *P* = 0.03^∗^: OR (95%CI) = 2.95 (1.10–7.920)) (Table 2).

Finally, comparison of allelic frequencies for the 14 bp variant showed a statistically significant difference between Del (21 in the HIV-uninfected EOPE and 18 in the HIV-infected EOPE) and Ins (5 in the HIV-uninfected EOPE and 20 in the HIV-infected EOPE) in the EOPE groups (Del/Ins, *P* = 0.009: OR (95%IC) = 4.66 (1.46–14.96)) (Table 3).

### 3.4. Genotypic Association of SNPs rs1707, rs1710, rs1063320, rs9380142, and rs1610696 for Preeclampsia and HIV

Testing the five SNPs for associations with PE and HIV within the normotensive group, we observed that three SNPs showed significant associations. The two SNPs not associating with HIV-infected compared to HIV-uninfected in the normotensive group are rs1710 and rs1063320 (Table 4). SNPs rs9380142, rs1610696, and rs1707 showed significant independent associations as follows: rs9380142 (AA *vs*. GG, *P* = 0.04^∗^: OR (95%CI) = 9.718 (0.5225 − 180.8); GG *vs*. GA, *P* = 0.059^∗^: OR (95%CI) = 216 (0.4712 − 180.3)), rs1610696 (CC *vs*. GC, *P* = 0.03^∗^: OR (95%CI) = 5.190 (1.006 − 26.78)), and rs1707 (TT *vs*. CC, *P* = 0.01^∗^: OR (95%CI) = 4.703 (1.239 − 17.85); CC *vs*. CT, *P* = 0.001^∗∗^: OR (95%CI) = 14.67 (2.430 − 88.53)) (Table 4).

Three sets of comparisons were performed within the PE group for the five SNPs, i.e., (i) HIV infected *vs*. HIV uninfected, (ii) HIV-infected EOPE *vs*. HIV-uninfected EOPE, and (iii) HIV-infected LOPE *vs*. HIV-uninfected LOPE. In the first set of comparisons, HIV infected *vs*. HIV uninfected in preeclamptic individuals, only two of the five SNPs showed an association with HIV and preeclampsia (Table 4): rs9380142 (AA *vs*. GG, *P* = 0.02^∗^: OR (95%CI) = 13.97 (0.73–269.4); AA *vs*. GA, *P* = 0.01^∗^: OR (95%CI) = 7.03 (1.38–35.81)) and rs1610696 with PE (CC *vs*. GG, *P* = 0.007^∗∗^: OR (95%CI) = 19.15 (0.98–374.0); GG *vs*. GC, *P* = 0.02^∗^: OR (95%CI) = 16.87 (0.81–352.1)). SNPs rs1710, rs1063320, and rs1707 showed no significant association.

In the second set of comparisons, HIV-infected EOPE *vs*. HIV-uninfected EOPE, only SNP rs1610696 showed an association with HIV status and early-onset preeclampsia (*P* = 0.04: OR (95%CI) = 10.04 (0.49 − 204.6)) (Table 4). While in the third set of comparisons, HIV-infected LOPE *vs*. HIV-uninfected LOPE, only SNP rs1710 showed no significant associations: rs1063320 (CC *vs*. GC, *P* = 0.04^∗^: OR (95%CI) = 11.47 (0.55–239.8)); rs9380142 (AA *vs*. GA, *P* = 0.03^∗^: OR (95%CI) = 13.00 (0.63–269.1)); and rs1610696 (CC *vs*. GG, *P* = 0.0003^∗∗∗^: OR (95%CI) = 31.87 (1.86–545.9); GG *vs*. GC, *P* = 0.0001^∗∗∗^: OR (95%CI) = 271.4 (12.07–50.41); CC *vs*. GC, *P* = 0.0009^∗∗∗^ and OR (95%CI) = 10.28 (0.17–69.471)). Finally, we have rs1707 (TT vs. CT, *P* = 0.0006^∗∗∗^ and OR (95%CI) = 40.00 (2.89–555.1)) (Table 4).

Normotensive HIV infected compared to preeclamptic HIV infected revealed two SNP associations, i.e., rs1610696 (GG *vs*. GC, *P* = 0.02^∗^: OR (95%CI) = 4.80 (1.16–19.93)) and rs1707, which significantly differed (TT *vs*. CC, *P* = 0.04^∗^: OR (95%CI) = 9.15 (0.51–163.5); CC *vs*. CT, *P* = 0.0007^∗∗∗^: OR (95%CI) = 49.28 (2.21–1098); TT *vs*. CT, *P* = 0.0092^∗∗^: OR (95%CI) = 5.96 (1.39–25.64)) (Table 4).

Furthermore, within the normotensive *vs*. preeclampsia groups, comparing the HIV-uninfected normotensive to HIV-uninfected preeclamptic individuals, there was a statistically significant difference in the association of the genotype frequencies of rs1610696 (CC *vs*. GG, *P* = 0.0003^∗∗∗^: OR (95%CI) = 31.87 (1.861 − 545.9); GG *vs*. GC, *P* = 0.0001^∗∗∗^: OR (95%CI) = 271.4 (12.07 − 6101); and CC *vs*. GC, *P* = 0.0009^∗∗∗^: OR (95%CI) = 10.28 (2.097 − 50.41)) (Table 4). The four remaining SNPs showed no significant associations (Table 4).

### 3.5. Allelic Association of SNPs rs1707, rs1710, rs1063320, rs9380142, and rs1610696 with Preeclampsia and HIV

Similar to the genotypic associations, for the allelic associations, three groups were compared across all SNPs, i.e., HIV status within normotensive, preeclamptic, and normotensive *vs*. preeclamptic groups. In the first group, HIV-infected *vs*. HIV-uninfected individuals who are normotensive showed only rs1707 as significant (T *vs*. C, *P* = 0.0072^∗∗^: OR (95%CI) = 5.313 (1.408 − 20.04)) (Table 5).

Three sets of comparisons were performed within the preeclampsia group for the five SNPs, i.e., (i) HIV infected *vs*. HIV uninfected, (ii) HIV-infected EOPE *vs*. HIV-uninfected EOPE, and (iii) HIV-infected LOPE *vs*. HIV-uninfected LOPE. In the first set of comparisons, HIV infected *vs*. HIV uninfected in preeclamptic individuals, only two of the five SNPs showed an association with HIV and preeclampsia, i.e., rs9380142 (A *vs*. G, *P* = 0.0003^∗∗∗^: OR (95%CI) = 10.72 (2.380 − 48.32)) and rs1610696 (C *vs*. G, *P* = 0.02^∗^: OR (95%CI) = 2.67 (1.12 − 6.38)) (Table 5).

In the second set of comparisons, HIV-infected EOPE *vs*. HIV-uninfected EOPE, only one SNP showed association, i.e., rs1610696 (C *vs*. G, *P* = 0.03^∗^: OR (95%IC) = 4.15 (1.05 − 16.50)) (Table 5). While in the third set of comparisons, HIV-infected LOPE *vs*. HIV-uninfected LOPE, only two SPNs showed association, i.e., rs9380142 (A *vs*. G, *P* = 0.02^∗^: OR (95%IC) = 9.00 (1.07 − 75.74)) and rs1707 (T *vs*. C, *P* = 0.001^∗∗^: OR (95%IC) = 20.50 (2.02 − 208.4)) (Table 5).

Normotensive HIV-infected compared to preeclamptic HIV-infected revealed one SNP association, i.e., rs1610696 (C *vs*. G, *P* = 0.02^∗^: OR (95%CI) = 9.000 (0.9813 − 82.54)) (Table 5).

Furthermore, within the normotensive *vs*. preeclampsia groups, comparing the HIV-uninfected normotensive to HIV-uninfected preeclamptic individuals, there was a statistically significant difference in the association of the allele frequencies of rs1610696 (C *vs*. G, *P* = 0.0001^∗∗∗^: OR (95%CI) = 21.91 (2.84 − 169.0)) (Table 5).

## 4. Discussion

The main findings of this study indicate that in preeclampsia, HLA-G 14 bp polymorphism (Ins/Ins and Del/Ins) and genotypic and allelic differences in rs9380142, rs1610696, and rs1707 are associated with HIV infection and preeclampsia in South African women of African ancestry.

We did not find any significant difference in the distribution of Del/Del in normotensive pregnant compared to PE groups. Similar results were shown by Larsen et al. in 2010 (*P* = 0.136) [30]. There was no association of Ins/Ins genotypic frequencies with PE development. Our findings demonstrated that Ins/Ins genotypic frequencies were higher in normotensive pregnant compared to PE women, and no allelic differences were noted. Our findings suggest that genotype (Ins/Ins) is not associated with the pathogenesis of PE. Nonetheless, the impact of HLA-G 14 bp polymorphism on the pathogenesis of PE is contradictory. Some studies have reported that there is no association between HLA-G 14 bp polymorphism with PE [46–49], while others have reported an association between HLA-G 14 bp polymorphism and PE [24, 50, 51]. In contrast to our findings, Emmery et al. in 2017 reported that children with the HLA-G 14 bp Ins/Ins genotype born to PE women with severe features have lower birthweight [52]. It is therefore plausible that the Ins/Ins genotype may be involved in the pathogenesis of PE. Similarly, the Ins/Ins genotype was shown to contribute to the development of high blood pressure in diabetes mellitus [53]. However, there is a paucity of studies reporting the association of this polymorphism in the development of PE; more studies with larger samples sizes are required.

Interestingly, we also demonstrate that the 14 bp Del/Ins polymorphism is associated with the development of PE. Del/Ins genotypic frequencies are present at higher frequency in PE compared to normotensive pregnant women. However, no allelic association was noted. Similar to our findings, a meta-analysis comparing the outcome across 14 studies highlighted that the 14 bp Del/Ins polymorphism was significantly linked to the risk of developing unexplained recurrent spontaneous miscarriage [54]. In contrast, Ferreira et al. in 2017 recently demonstrated that the maternal HLA-G 14 bp Del/Ins polymorphism is not associated with PE risk in Brazilian women [55]. Interestingly several previous studies conducted on African American, Chinese, Indian, Irish, French, and Norwegian women reported a similar nonassociation of this polymorphism with PE development [26, 28, 29, 46, 56].

Only the Ins/Ins polymorphism showed an association with HIV infection. There was an overrepresentation of this genotype between the HIV-infected normotensive compared to uninfected normotensive groups; however, these findings were not significant. Interestingly, we noted a significant difference between preeclamptic groups. This genotype was expressed higher in HIV-infected PE compared to uninfected PE. Our findings suggest that this polymorphism is associated with the pathophysiology of HIV infection, rather than PE.

Similar to our findings, a study by da Silva et al. in 2014 reported higher Ins genotype among African-derived HIV-infected patients which also indicated that Ins is associated with the progression of HIV [57].

Contradictory to our findings, a study in Zambia showed that Ins allele frequencies are higher in HIV-exposed uninfected infants compared to HIV-infected infants [58]. The 14 bp insertion polymorphism has been reported to vary in frequency between populations, ranging from 12% in Japanese to 32% in Europeans, and up to 43% in Africans and also within populations [59, 60]; variation within the population may account for the different results obtained in our study from that obtained in the Zambian study. However, more studies on this genotype are recommended in order to validate these findings.

The SNP 3022 (rs1707) has been previously reported in unsuccessful pregnancies such as recurrent pregnancy loss [18, 61] and spontaneous preterm births [62]. In the current study, we found higher CT genotypic frequencies in PE, more especially LOPE compared to normotensive pregnant women. Our findings are in conflict with studies that have reported no differences in distribution of the rs1707 genotype between the control and PE groups [18, 30, 62].

The rs1707 polymorphism has been previously reported to be involved in the pathophysiology of HIV infection [63]. In our study, the genotypic comparison TT *vs*. CT differed between the HIV-uninfected LOPE and HIV-infected LOPE. CT was higher in infected LOPE compared to uninfected LOPE. We did not find any allelic association of rs1707 with HIV infection, although a previous study has reported a T allele association of this polymorphism with HIV infection compared to controls [63]. This is the first study to associate this polymorphism with HIV infection-associated pregnancies. More studies are needed in order to confirm our findings.

There was no statistical significance in comparison of SNP 3029 (rs1710) genotypic frequencies as well as allelic frequencies across the study groups. The GG genotype and the G allele were equally distributed across all groups. Our findings are corroborated by similar findings of Larsen et al. in 2010 who also reported no statistically significant difference in the distribution of rs1710 genotypes between control and PE [30]. To the best of our knowledge, this is the first study to report on this SNP based on HIV infection. Although we noted no significant difference between HIV-infected and uninfected groups, more studies are still needed in order to validate our findings.

SNP 3161 (rs1063320) has been previously associated with PE development [49]. In our study, based on pregnancy type, no significant genotypic and allelic association of the rs1063320 polymorphism was observed. Similarly, various other studies report no association of this polymorphism with PE [18, 30, 62].

We observed no genotypic and allelic association of SNP 3161 (rs1063320) with HIV infection in PE groups. Our findings showed that the CC genotype was higher in HIV-uninfected LOPE compared to infected LOPE. It is therefore plausible that this SNP is not involved in the pathogenesis of HIV infection; moreover, preeclamptic women presenting with this genotype are likely to be HIV uninfected. Our findings are in accordance with that of da Silva et al. in 2014 who demonstrated higher CC genotypic frequencies in an HIV-uninfected African population [57]. However, more studies are needed to confirm these discrepancies.

Comparison between the normotensive pregnant and PE groups showed no statistically significant difference in the distribution of the genotypes and alleles of SNP 3206 (rs9380142) across the groups. This finding is consistent with another study that reports no significant difference across normotensive versus preeclamptic women [30] as well as between normotensive versus recurrent pregnancy loss [18]. However, the AA genotype occurs more frequently in both PE and control groups but is not statistically different across the groups [62]. Speculations are that “a G/A SNP at position +3187 (rs9380142)” may be associated with influencing the stability of mRNA and thus affecting the expression of sHLA-G [32]. In contrast to our findings, a study by Yie et al. in 2008 reported a significant association of rs9380142 with risk of preeclampsia. A significant overrepresentation of an A/A genotype at this SNP locus in offspring from preeclamptic cases compared with controls has been reported [64].

SNP 3206 (rs9380142) has been previously associated with HIV infection [65]. In our study, the AA genotype distribution was significantly different between HIV-uninfected and infected PE groups. These genotypes occur more in the HIV-infected PE group compared to the HIV-uninfected group. It was also found that the A and G allele distribution differs significantly in the HIV-uninfected and HIV-infected PE groups. The A allele occurred more frequently in HIV-infected PE, more specifically LOPE compared to HIV-uninfected PE, than the G allele in these groups. These findings indicate that HIV-infected women with the AA genotype are likely to develop preeclampsia, particularly the LOPE group. To the best of our knowledge, this is the first study reporting on the rs9380142 polymorphism in HIV-associated preeclamptic pregnancy. This still needs verification. Our findings are in contrast with that of Hong et al. in 2015 who observed more GG genotypes to be associated with *in utero* mother-to-child transmission of HIV compared to HIV nontransmitting women [65].

In the current study, the results show that all the three genotypes in SNP 3215 (rs1610696) distribution significantly varied between the normotensive and PE women. The frequency of the genotype CC was higher in PE compared to normotensive pregnant women. Also, the allelic frequency significantly was different between normotensive and PE women. The C allele of this SNP was higher in PE compared to normotensive women. This finding alludes to the fact that this polymorphism is involved in the pathogenesis of PE. Our findings are in support with Lee et al. who in 2018 found more association of the C genotype in PE compared to normotensive pregnant women [62]. Contradictory findings show no association difference between the genotypic distribution of rs1610696 in controls and PE [18, 30, 62].

We noticed a strong genotypic association of the SNP 3215 (rs1610696) polymorphism with HIV infection. GG was higher in the HIV-infected PE more especially the LOPE groups compared to uninfected PE. Our findings suggest that HIV pregnant women presenting the GG genotype are at risk of developing PE, more especially LOPE. The mechanism behind this is not understood and to the best of our knowledge, this is the first study to report on the association of this SNP with HIV infection in pregnant women. A similar study by Hong et al. in 2015 demonstrated no association of this SNP with HIV infection [65].

## 5. Strengths and Limitations of This Study

To the best of our knowledge, this is the first study to examine HLA-G polymorphisms based on pregnancy type and HIV status in a South African population of African ancestry. Of note, our data is limited by a small sample size due to the use of archived samples. Another limitation was the ethnicity of women. In South Africa, PE has the highest prevalence in the province of KwaZulu-Natal (12%), occurring predominantly in primigravidae [66]. Furthermore, this study utilizes retrospectively collected samples, and therefore, the number of samples was limited. Importantly, the six SNPs selected in this study do not reflect the total SNPs available for HLA-G.

## 6. Conclusion

This study has shown that HIV infection may be associated with selected HLA-G gene polymorphisms and the risk of preeclampsia in HIV-infected South Africans of African ancestry. This study has shown that the 14 bp genotype in PE may vary significantly due to HIV infection. Also, this study has shown that the genotypic and allelic frequencies vary across the different SNPs studied taking into consideration HIV infection. This study opens up an area that requires further research to confirm the impact of immunodeficient states on the gene polymorphism and the development of preeclampsia in HIV-associated preeclamptic pregnancies taking into consideration HAART treatment as the primary source of predisposition.

## Figures and Tables

**Table 1:** Patient demographics of the study groups (normotensive = 134; early − onset preeclampsia = 32; late − onset preeclampsia = 27).

Variables	Groups	Median	Q1-Q3	Mean ± SD	*P* value
Maternal weight (kg)	N	77	65-100	81.92 ± 18.35	
	EOPE	79	67.50-100.5	86.85 ± 30.31	0.1316
	LOPE	96.50	72.50-113.0	93.15 ± 21.95	
Maternal height (m)	N	157	153.25-163	157.5 ± 7.242	
	EOPE	159	154.5-164	158.8 ± 7.967	0.6761
	LOPE	160	155-164	159.1 ± 6.934	
BMI (kg/m^2^)	N	32.05	25.72-38.65	32.59 ± 7.301	
	EOPE	31.64	25.80-39.78	33.52 ± 9.483	0.0638
	LOPE	38	32.93-41.50	37.20 ± 8.021	
Systolic blood pressure (mmHg)	N	109	98.25-113.75	108.0 ± 11.25	
	EOPE	146	144-157	149.9 ± 10.17	<0.0001^∗∗∗^
	LOPE	145	140-149.75	145.40 ± 7.35	
Diastolic blood pressure (mmHg)	N	65.5	61-72	65.52 ± 9.38	
	EOPE	95	90-104	96.70 ± 9.20	<0.0001^∗∗∗^
	LOPE	94	90-98	93.25 ± 5.87	
Gestational age (weeks)	N	35	26-38	31.88 ± 6.73	<0.0001^∗∗∗^
	EOPE	24	20-30	24.25 ± 5.77	
	LOPE	36	35-37.25	35.95 ± 1.96	
Maternal age (years)	N	28	25-32.75	28.60 ± 5.90	
	EOPE	28.5	22.75-34.25	28.19 ± 7.35	0.9574
	LOPE	29	24-32.5	28.45 ± 7.13	

N: normotensive; EOPE: early-onset preeclampsia; LOPE: late-onset preeclampsia. Asterisks denote significance: ^∗^*P* < 0.05, ^∗∗^*P* < 0.01, and ^∗∗∗^*P* < 0.001.

**Table 2:** 14 bp genotypic frequencies.

	Normotensive			Preeclampsia									Normotensive *vs*. preeclampsia	
Polymorphisms	HIV- (*n* = 74)	HIV+ (*n* = 60)	HIV- *vs*. HIV+ OR (95%), *P* value	HIV- (*n* = 34)	HIV+ (*n* = 25)	HIV- *vs*. HIV+ OR (95%), *P* value	HIV- EOPE (*n* = 13)	HIV+ EOPE (*n* = 19)	HIV- *vs*. HIV + OR (95%), *P* value	HIV- LOPE (*n* = 21)	HIV+ LOPE (*n* = 6)	HIV- LOPE *vs*. HIV+ LOPE OR (95%), *P* value	HIV+ normotensive *vs*. HIV+ PE OR (95%), *P* value	HIV- normotensive *vs*. HIV- PE OR (95%), *P* value
Del/Del Del/Del *vs*. Ins/Ins	33 (44.59%)	20 (33.33%)	2.567 (0.97–1.84) *P* = 0.06	14 (41.17%)	7 (28%)	25.13 (1.24–509.5) *P* = 0.004^∗^	8 (61.53%)	5 (26.31%)	20.09 (0.93–433.1) *P* = 0.01^∗^	6 (28.57%)	2 (33.33%)	—	1.22 (0.34–1.95) *P* = 0.76	8.22 (0.45–151.0) *P* = 0.06
Ins/Ins Ins/Ins *vs*. Del/Ins	9 (12.16%)	14 (23.33%)	1.92 (0.91–1.59) *P* = 0.192	0 (0%)	6 (24%)	21.32 (1.71–4.17) *P* = 0.005^∗∗^	0 (0%)	6 (31.57%)	8.41 (0.39–181.3) *P* = 0.08	0 (0%)	0 (0%)	—	1.08 (0.33–3.49) *P* = 0.82	13.44 (0.722–249.9) *P* = 0.02^∗^
Del/Ins Del/Del *vs*. Del/Ins	32 (43.24%)	26 (43.33%)	1.34 (0.63–2.87) *P* = 0.45	20 (58.82%)	12 (48%)	1.20 (0.37–3.81) *P* = 0.45	5 (38.46)	8 (42.10%)	2.50 (0.53–12.44) *P* = 0.24	15 (71.42%)	4 (66.66%)	1.25 (0.18–8.73) *P* = 0.82	1.32 (0.44–3.96) *P* = 0.62	2.95 (1.10–7.920) *P* = 0.03^∗^

Asterisks denote significance: ^∗^*P* < 0.05, ^∗∗^*P* < 0.01, and ^∗∗∗^*P* < 0.001. Del=deletion, Ins=insertion, HIV-=HIV uninfected, HIV+=HIV infected, EOPE=early-onset preeclampsia, and LOPE=late-onset preeclampsia.

**Table 3:** 14 bp allelic frequencies.

	Normotensive			Preeclampsia									Normotensive *vs*. preeclampsia	
Polymorphisms	HIV- (*n* = 74)	HIV+ (*n* = 60)	HIV- *vs*. HIV+ OR (95%), *P* value	HIV- (*n* = 34)	HIV+ (*n* = 25)	HIV- *vs*. HIV+ OR (95%), *P* value	HIV- EOPE (*n* = 13)	HIV+ EOPE (*n* = 19)	HIV- *vs*. HIV+ OR (95%), *P* value	HIV- LOPE (*n* = 21)	HIV+ LOPE (*n* = 6)	HIV- LOPE *vs*. HIV+ LOPE OR (95%), *P* value	HIV+ normotensive *vs*. HIV+ PE OR (95%), *P* value	HIV- normotensive *vs*. HIV- PE OR (95%), *P* value
Del	98 (66.21%)	66 (55%)	1.60 (0.98–2.63) *P* = 0.08	48 (70.58%)	26 (59.09%)	1.66 (0.75–3.68) *P* = 0.23	21 (80.76%)	18 (47.36%)	4.66 (1.46–14.96) *P* = 0.009^∗∗^	27 (64.28%)	8 (66.66%)	1.11 (0.29–4.31) *P* = 1.00	1.18 (0.59–2.38) *P* = 0.73	1.22 (0.66–2.28) *P* = 0.64
Ins	50 (33.78%)	54 (45%)		20 (29.41%)	18 (40.90%)		5 (19.23%)	20 (52.63%)		15 (35.71%)	4 (33.33%)			

Asterisks denote significance: ^∗^*P* < 0.05, ^∗∗^*P* < 0.01, and ^∗∗∗^*P* < 0.001. Del=deletion, Ins=insertion, HIV-=HIV uninfected, HIV+=HIV infected, EOPE=early-onset preeclampsia, and LOPE=late-onset preeclampsia.

**Table 4:** Genotype frequencies.

	Normotensive			Preeclampsia									Normotensive *vs*. preeclampsia	
Polymorphisms	HIV- (*n* = 74)	HIV+ (*n* = 60)	HIV- *vs*. HIV+ OR (95%), *P* value	HIV- (*n* = 34)	HIV+ (*n* = 25)	HIV- *vs*. HIV+ OR (95%), *P* value	HIV- EOPE (*n* = 13)	HIV+ EOPE (*n* = 19)	HIV- *vs*. HIV+ OR (95%), *P* value	HIV- LOPE (*n* = 21)	HIV+ LOPE (*n* = 6)	HIV- LOPE *vs*. HIV+ LOPE OR (95%), *P* value	HIV+ normotensive *vs*. HIV+ PE OR (95%), *P* value	HIV- normotensive *vs*. HIV- PE OR (95%), *P* value
SNP 3022 (rs1707)														
TT TT *vs*. CC	59 (79.72%)	46 (76.66%)	4.703 (1.239-17.85) *P* = 0.01^∗^	28 (82.35%)	18 (72%)	10.00 (0.4369-228.9) *P* = 0.09	9 (69.23%)	12 (63.15%)	1.778 (0.09877-32.00) *P* = 0.69	19 (90.47%)	2 (33.33%)	10.00 (0.4369-228.9) *P* = 0.09	9.15 (0.51-163.5) *P* = 0.04^∗^	3.471 (0.1734-69.47) *P* = 0.22
CC CC *vs*. CT	3 (4.05%)	11 (18.33%)	14.67 (2.430-88.53) *P* = 0.001^∗∗^	1 (2.94%)	1 (4%)	1.400 (0.06965-28.14) *P* = 0.83	1 (7.69%)	1 (5.26%)	1.333 (0.05708-31.15) *P* = 0.86	1 (4.76%)	1 (16.66%)	4.000 (0.1167-137.1) *P* = 0.43	49.29 (2.21-1098) *P* = 0.0007^∗∗∗^	3.080 (0.1348-70.39) *P* = 0.28
CT TT *vs*. CT	12 (16.21%)	3 (5%)	3.119 (0.8307-11.71) *P* = 0.08	5 (14.70%)	6 (24%)	2.256 (0.6210-8.193) *P* = 0.21	3 (23.07%)	6 (31.57%)	2.370 (0.4306-13.05) *P* = 0.31	1 (4.76%)	3 (50%)	40.00 (2.89-555.1) *P* = 0.0006^∗∗∗^	5. (1.39-25.64) *P* = 0.0092^∗∗^	1.180 (0.3794-3.668) *P* = 0.78
SNP 3029 (rs1710)														
GG GG *vs*. CC	30 (40.54%)	26 (43.33%)	1.212 (0.5406-2.715) *P* = 0.64	17 (50%)	13 (52%)	1.162 (0.3517-3.842) *P* = 0.80	7 (53.84%)	9 (47.36%)	1.556 (0.3286-7.364) *P* = 0.58	10 (47.61%)	3 (50%)	4.714 (0.2125-104.6) *P* = 0.53	1.313 (0.4583-3.759) *P* = 0.61	1.259 (0.4695-3.377) *P* = 0.65
CC CC *vs*. GC	20 (27.02%)	21 (35%)	1.938 (0.7791-4.823) *P* = 0.15	9 (26.47%)	8 (32%)	1.778 (0.3840-8.231) *P* = 0.46	4 (30.76%)	8 (42.10%)	2.000 (0.2008-19.93) *P* = 0.55	5 (23.80%)	1 (16.66%)	4.231 (0.1653-108.3) *P* = 0.22	1.238 (0.3097-4.949) *P* = 0.76	1.350 (0.4394-4.147) *P* = 0.60
GC GG *vs*. GC	24 (32.43%)	13 (21.66%)	1.600 (6802-3.764) *P* = 0.28	8 (23.52%)	4 (16%)	1.529 (0.3767-6.209) *P* = 0.55	2 (15.38%)	2 (10.52%)	1.286 (0.1431-11.55) *P* = 0.82	6 (28.57%)	2 (33.33%)	1.200 (0.1662-8.663) *P* = 0.86	1.625 (0.4412-5.985) *P* = 0.46	1.700 (0.6270-4.609) *P* = 0.29
SNP 3161 (rs1063320)														
GG GG *vs*. CC	22 (29.72%)	25 (41.66%)	1.705 (0.6369-4.562) *P* = 0.29	10 (29.41%)	8 (32%)	2.667 (0.543-13.09) *P* = 0.23	4 (30.76%)	8 (42.10%)	2.000 (0.2704-14.79) *P* = 0.49	6 (28.57%)	1 (16.66%)	1.167 (0.05930-22.95) *P* = 0.92	1.067 (0.2341-4.860) *P* = 0.93	1.467 (0.4905-4.385) *P* = 0.49
CC CC *vs*. GC	15 (20.27%)	10 (16.66%)	1.014 (0.3929-2.615) *P* = 0.98	10 (29.41%)	3 (12%)	3.333 (0.7527-14.76) *P* = 0.10	3 (23.07%)	3 (15.78%)	1.333 (0.1956-9.087) *P* = 0.77	7 (33.33%)	1 (16.66%)	11.47 (0.5486-239.8) *P* = 0.04^∗^	1.867 (0.4392-7.934) *P* = 0.39	1.762 (0.6421-4.835) *P* = 0.27
GC GG *vs*. GC	37 (50%)	25 (41.66%)	1.682 (0.7822-3.616) *P* = 0.18	14 (41.17%)	14 (56%)	1.250 (0.3806-4.105) *P* = 0.71	6 (46.15%)	8 (42.10%)	1.500 (0.3026-7.435) *P* = 0.62	8 (38.09%)	4 (66.66%)	9.941 (0.4693-210.6) *P* = 0.05	1.750 (0.6243-4.905) *P* = 0.28	1.201 (0.4562-3.163) *P* = 0.71
SNP 3206 (rs9380142)														
AA AA *vs*. GG	51 (68.91%)	45 (75%)	9.718 (0.5225-180.8) *P* = 0.04^∗^	18 (52.94%)	22 (88%)	13.97 (0.73-269.4) *P* = 0.02^∗^	9 (69.23%)	16 (84.21%)	1.700 (0.09538-30.30) *P* = 0.72	8 (38.09%)	4 (66.66%)	8.412 (0.3902-181.3) *P* = 0.08	1.957 (0.1169-32.75) *P* = 0.63	2.833 (0.7335-10.94) *P* = 0.12
GG GG *vs*. GA	5 (6.75%)	1 (1.66%)	9.216 (0.4712-180.3) *P* = 0.05^∗^	5 (14.70%)	1 (4%)	2.391 (0.09722-58.82) *P* = 0.87	1 (7.69%)	1 (5.26%)	1.500 (0.05533-40.67) *P* = 0.80	5 (23.80%)	1 (16.66%)	1.600 (0.08052-31.79) *P* = 0.76	7.500 (0.3244-173.4) *P* = 0.16	1.636 (0.3841-6.971) *P* = 0.50
GA AA *vs*. GA	18 (24.32%)	14 (23.33%)	1.059 (0.4786-2.342) *P* = 0.89	11 (32.35%)	2 (8%)	7.03 (1.38-35.81) *P* = 0.01^∗^	3 (23.07%)	2 (10.52%)	2.550 (0.3618-17.97) *P* = 0.34	8 (38.09%)	1 (16.66%)	13.00 (0.63-269.1) *P* = 0.03^∗^	3.833 (0.8063-18.22) *P* = 0.07	1.731 (0.6880-4.358) *P* = 0.24
SNP 3215 (rs1610696)														
CC CC *vs*. GG	43 (58.10%)	29 (48.33%)	1.227 (0.5989-2.514) *P* = 0.58	23 (67.64%)	13 (52%)	19.15 (0.98-374.0) *P* = 0.0070^∗∗^	10 (76.92%)	11 (57.89%)	10.04 (0.49-204.6) *P* = 0.04^∗^	13 (61.90%)	2 (33.33%)	15.51 (1.98-121.4) *P* = 0.0009^∗∗∗^	2.152 (0.6712-6.898) *P* = 0.19	31.87 (1.861-545.9) *P* = 0.0003∗^∗∗^
GG GG *vs*. GC	29 (39.18%)	24 (40%)	4.229 (0.8022-22.29) *P* = 0.07	1 (2.94%)	5 (20%)	16.87 (0.81-352.1) *P* = 0.02^∗^	1 (7.69%)	5 (26.31%)	11.00 (0.4253-284.5) *P* = 0.06	1 (4.76%)	1 (16.66%)	159.5 (13.10-1942) *P* < 0.0001^∗∗∗^	4.800 (1.16-19.93) *P* = 0.02^∗^	271.4 (12.07-6101) *P* < 0.0001^∗∗∗^
GC CC *vs*. GC	2 (2.70%)	7 (11.66%)	5.190 (1.006-26.78) *P* = 0.03^∗^	10 (29.41%)	7 (28%)	1.126 (0.3506-3.616) *P* = 0.84	2 (15.38%)	3 (15.78%)	1.100 (0.1790-6.758) *P* = 0.92	7 (33.33%)	3 (50%)	3.25 (0.4799-22.01) *P* = 0.21	2.231 (0.6485-7.673) *P* = 0.19	10.28 (2.097-50.41) *P* = 0.0009^∗∗∗^

Asterisks denote significance: ^∗^*P* < 0.05, ^∗∗^*P* < 0.01, and ^∗∗∗^*P* < 0.001. SNP=single nucleotide polymorphisms, Del=deletion, Ins=insertion, HIV-=HIV uninfected, HIV+=HIV infected, EOPE=early-onset preeclampsia, and LOPE=late-onset preeclampsia.

**Table 5:** Allelic frequencies.

	Normotensive			Preeclampsia									Normotensive *vs*. preeclampsia	
Polymorphisms	HIV- (*n* = 74)	HIV+ (*n* = 60)	HIV- *vs*. HIV+ OR (95%), *P* value	HIV- (*n* = 34)	HIV + (*n* = 25)	HIV- *vs*. HIV+ OR (95%), *P* value	HIV- EOPE (*n* = 13)	HIV+ EOPE (*n* = 19)	HIV- *vs*. HIV+ OR (95%), *P* value	HIV- LOPE (*n* = 21)	HIV+ LOPE (*n* = 6)	HIV- LOPE *vs*. HIV+ LOPE OR (95%), *P* value	HIV+ normotensive *vs*. HIV+ PE OR (95%), *P* value	HIV- normotensive *vs*. HIV- PE OR (95%), *P* value
SNP 3022 (rs1707)														
T T *vs*. C	130 (87.83%)	95 (79.16%)	5.313 (1.408-20.04) *P* = 0.0072^∗∗^	61 (89.70%)	42 (84%)	2.051 (0.6106-6.890) *P* = 0.24	21 (80.76%)	30 (78.94%)	2.121 (0.4328-10.40) *P* = 0.35	39 (92.85%)	7 (58.33%)	20.50 (2.02-208.4) *P* = 0.001^∗∗^	1.617 (0.6491-4.026) *P* = 0.30	1.745 (0.6193-4.915) *P* = 0.23
G G *vs*. C	84 (56.75%)	65 (54.16%)	1.111 (0.6841-1.803) *P* = 0.67	42 (61.76%)	30 (60%)	1.077 (0.5097-2.275) *P* = 0.85	16 (61.53%)	20 (52.63%)	1.108 (0.4104-2.990) *P* = 0.84	26 (61.90%)	8 (66.66%)	1.538 (0.4124-5.739) *P* = 0.52	1.269 (0.6493-2.481) *P* = 0.49	1.231 (0.6840-2.215) *P* = 0.49
SNP 3161(rs1063320)														
G G *vs*. C	81 (54.72%)	75 (62.5%)	1.379 (0.8434-2.253) *P* = 0.19	34 (50%)	30 (60%)	1.500 (0.7163-3.141) *P* = 0.28	14 (53.84%)	24 (63.15%)	1.440 (0.5219-3.974) *P* = 0.50	20 (47.61%)	6 (50%)	1.538 (0.4124-5.739) *P* = 0.52	1.111 (0.5652-2.184) *P* = 0.76	1.209 (0.6801-2.149) *P* = 0.52
SNP 3206 (rs9380142)														
A A *vs*. G	120 (81.08%)	104 (86.66%)	1.633 (0.8276-3.223) *P* = 0.15	47 (69.11%)	46 (92%)	10.72 (2.380-48.32) *P* = 0.0003^∗∗∗^	21 (80.76%)	34 (89.47%)	2.348 (0.3638-15.15) *P* = 0.36	24 (57.14%)	9 (75%)	9.00 (1.07-75.74) *P* = 0.02^∗^	3.429 (0.7538-15.59) *P* = 0.09	1.915 (0.9909-3.701) *P* = 0.05
SNP 3215 (rs1610696)														
C C *vs*. G	88 (59.45%)	65 (54.16%)	1.221 (0.7512-1.984) *P* = 0.42	56 (82.35%)	33 (66%)	2.67 (1.12-6.38) *P* = 0.02^∗^	22 (84.61%)	25 (65.78%)	4.15 (1.05-16.50) *P* = 0.03^∗^	33 (78.57%)	7 (58.33%)	1.500 (0.3544-6.349) *P* = 0.58	9.000 (0.9813-82.54) *P* = 0.02^∗^	21.91 (2.84-169.0) *P* < 0.0001^∗∗∗^

Asterisks denote significance: ^∗^*P* < 0.05, ^∗∗^*P* < 0.01, and ^∗∗∗^*P* < 0.001. SNP=single nucleotide polymorphisms, Del=deletion, Ins=insertion, HIV- =HIV uninfected, HIV+ =HIV infected, EOPE=early-onset preeclampsia, and LOPE=late-onset preeclampsia.

## Data Availability

The data used to support the findings of this study are available from the corresponding author upon request.
